# Warriors and Peacekeepers: Testing a Biosocial Implicit Leadership Hypothesis of Intergroup Relations Using Masculine and Feminine Faces

**DOI:** 10.1371/journal.pone.0030399

**Published:** 2012-01-20

**Authors:** Brian R. Spisak, Peter H. Dekker, Max Krüger, Mark van Vugt

**Affiliations:** 1 Department of Social and Organizational Psychology, Vrije Universiteit (VU) University Amsterdam, Amsterdam, The Netherlands; 2 Institute for Cogntive and Evolutionary Anthropology, University of Oxford, Oxford, United Kingdom; Royal Holloway, University of London, United Kingdom

## Abstract

This paper examines the impact of facial cues on leadership emergence. Using evolutionary social psychology, we expand upon implicit and contingent theories of leadership and propose that different types of intergroup relations elicit different implicit cognitive leadership prototypes. It is argued that a biologically based hormonal connection between behavior and corresponding facial characteristics interacts with evolutionarily consistent social dynamics to influence leadership emergence. We predict that masculine-looking leaders are selected during intergroup conflict (war) and feminine-looking leaders during intergroup cooperation (peace). Across two experiments we show that a general categorization of leader versus nonleader is an initial implicit requirement for emergence, and at a context-specific level facial cues of masculinity and femininity contingently affect war versus peace leadership emergence in the predicted direction. In addition, we replicate our findings in Experiment 1 across culture using Western and East Asian samples. In Experiment 2, we also show that masculine-feminine facial cues are better predictors of leadership than male-female cues. Collectively, our results indicate a multi-level classification of context-specific leadership based on visual cues imbedded in the human face and challenge traditional distinctions of male and female leadership.

## Introduction

Leadership is a universal feature of human social life. It is present in all known cultures [1], and it is relevant for many key human group activities including matters of warfare and peacekeeping within and between groups [2]. Despite the plethora of findings, we know very little about the evolutionary origins and functions of leadership [3]. For example, do different leadership prototypes emerge in different fitness-relevant situations? Why is there (still) male bias in leadership, and is there a niche for more feminine leadership?

The current research adopts an evolutionary social psychological approach to examine potential masculine-feminine categorization biases during leadership emergence. We argue that different fitness-relevant intergroup challenges elicit different implicit cognitive leader prototypes that are ultimately grounded in our biology. We suggest that these masculine-feminine cognitive leadership prototypes are highly automatic and emerged to deal with key human intergroup challenges. In addition, the current research attempts to align itself with previous implicit leadership theories by highlighting levels of categorization from a broad leader/nonleader distinction to the context-specific differences of masculinity and femininity.

### Leadership and the Benefits of Social Coordination

Leadership is broadly defined as the ability to coordinate the activities of individuals to achieve mutual goals [4]. Evolutionary thinking also requires consideration of why leadership emerges spontaneously in human groups and what its ultimate functions are [5]. Specifically, we know that humans are among a number of species that have evolved a group living strategy because cohesive groups increase reproductive opportunities [6], [7], and leadership adds to this social cohesion by coordinating group activities in the face of various challenges such as warfare, peacekeeping, or resource scarcity [3]. A key adaptive challenge then is for group members to identify an individual to follow in any particular situation [5].

This pressure for situational leadership forms the foundation of what has been termed the biosocial contingency model of leadership [8] – an evolutionary-based extension of traditional contingent leadership theory [9]. Essentially, shifting situation requirements (e.g., conflict or cooperation) interact with biologically-based individual differences (e.g., masculine or feminine cues) to contingently select for group members with the most appropriate context-specific traits to lead. Those individuals that closely match the prototype will attract followers. For instance, a time of conflict will likely select for leaders displaying more dominant and aggressive signals. Interestingly, contingent leadership is something we share with a variety of social species ranging from geese and cattle, to more advanced primates [10]–[13], suggesting that the mechanism is an evolved feature of sociality.

**Figure 1:**
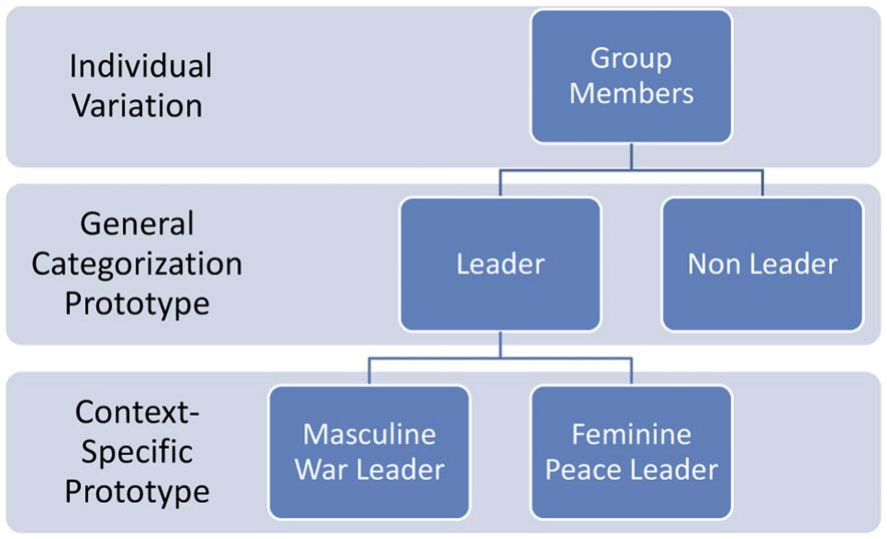
Biosocial leadership categorization: example from group-level variation to context-specific cognitive leadership prototype.

How then has this shared “leadership” mechanism evolved in human groups to address intergroup relations? We suggest the formation of cognitive heuristics for identifying individuals that closely match the prototype of either a war or peace leader. This is akin to implicit leadership theories [14], [15] which propose people hold broad to increasingly distinct categorical perceptions of what good leaders look like and how they behave, and the likelihood of leader emergence depends on the match between an individual's features and the prevailing leadership prototype. We also support this notion of categorization and likewise suspect potential leaders *do* share a perceived common threshold of general leadership traits and subsequently, passing this initial evaluation, differ at a context-specific level of leadership. However, expanding upon this categorization approach, a biosocial framework is incorporated to glean a deeper understanding of leadership prototype formation (see Fig. 1).

Throughout mammalian evolution it appears an innate ability to form and act upon prototypes has been selected for - such as the automatic fear response to objects resembling a snake [16]. In human groups this built-in mechanism may have been co-opted for increasing the efficiency of selecting context-specific leaders. Various cognitive-based theories find this sort of prototypical heuristic processing to be an integral part of our innate bounded rationality [17], [18]. For instance, assuming that intergroup relations are fitness-relevant, those individuals who carried the wrong impression of who to follow (or did not follow at all) would likely disappear from the gene pool, leaving only those individuals (or groups) that could correctly assign prestige to those leaders who were most likely to increase reproductive success. This is similar to the selection pressure for correctly assessing and prototyping physical formidability amongst individuals in competitive social groups [19]. Further, fMRI research has shown that competition and cooperation initiate automatic arousal and occupy distinct neurological regions [20], and perhaps activation of these separate areas is in part an adaptive response to initiate context-specific decision rules about which prototype to follow.

How then, in human evolutionary history, has the pressure of managing intergroup relations shaped these prototypes? Although for much of human history population densities have been much lower than today, early humans would have had regular encounters with members of outgroups which either presented a threat or opportunity, and intergroup interactions would have oscillated between open hostility and peaceful co-existence [21]. Archaeological evidence suggests that intergroup conflict was lethal (potentially accounting for 20–30% of ancestral male deaths) [22] and frequent enough to alter human social behavior [23]. Data from modern hunter-gatherer societies confirms that “raiding and trading” are fairly typical intergroup behaviors [24], [25]. Finally, large-scale cooperation (including with non-kin) has likely been practiced throughout human evolution [26], and there is evidence from traditional societies that different individuals took on leadership roles when cooperatively engaging in war or peace [27].

This combination of interdisciplinary evidence dictates that humans have potentially evolved a suite of cognitive adaptations to manage and exploit intergroup relations [28]. This would likely include decision rules to determine which individuals to follow as leaders across different intergroup settings [29]. Because the skills to attack, dominate, and exploit other groups are very different from the skills to foster or maintain peaceful relations we suspect different leader prototypes to pop-up in war or peace time.

### Facial Masculinity and Femininity as Cognitive Leadership Prototypes

Interestingly, these differentiated constellations of skills required for competition or cooperation seem to parallel phenotypic associations with hormones such as testosterone and estrogen. For instance, higher levels of testosterone are associated with masculine facial features (e.g., stronger jaw and brow, and thinner eyes) [30], [31], and behaviorally they are associated with dominance behavior, risk-taking, and status-seeking [32]–[34]. In contrast, estrogen underlies feminine facial features such as larger eyes and fuller lips [31] and estrogen is associated behaviorally with more tending-and-befriending [35]. Thus, it can be deduced that warfare elicits a masculine-looking prototype and peacekeeping a feminine-looking prototype and this implicit contingency will affect voting preferences.

The heuristic decision rule for determining such situation-based leadership could be quite simple. We suspect that followership investment (i.e., the investment of energy, resources, authority, votes, and so on in a leader for a common goal) [36] during intergroup relations is in part driven by a rule such as: “*if* conflict leadership *then* masculine-looking, *if* cooperative leadership *then* feminine-looking.” Further, as followership perception of the intergroup environment shifts, so too does followership investment in a particular leader according to the implicit rule.

Previous research has shown that perceived variations of facial features such as competence can predict the outcome of actual political elections [37]. Likewise, recent research on self-resembling opposite-sex faces suggests that visual attribution mechanisms may, at times, rely on context-based experience for activation [38], and priming individuals with specific group goals can alter specific facial preferences [39]. Thus, for instance, considering that masculinized male faces are perceived to be both more socially and physically dominant [40] it is likely this facial prototype will be preferred when a context of conflict is activated.

In fact, it has been shown that asking people about whom they would prefer as leader during “war” or “peace” elicits a preference for a more masculine- or feminine-looking male face, respectively [41]. Morphing the facial features of the more masculine-looking George Bush and the more feminine-looking John Kerry (i.e., the 2004 United States Presidential candidates) on an unrecognizable male base-face they replicated the result. Here we extend this work by examining if an implicit facial categorization process exists for first identifying a general prototype of leadership (i.e., leader/nonleader). We also investigate how intergroup context contingently influences preferences for a masculine or feminine leader prototype. Furthermore, we look at whether these prototypes remain consistent across Western and Eastern cultures. Finally, we investigate if a hormonally-based variation of facial masculinity-femininity is a better predictor of leadership preferences than biological sex (i.e., male or female) of presidential candidates in mock elections during war and peace.

It is important to note that on average men have higher levels of testosterone than women, and conversely for estrogen. This is likely a reflection of the differentiated benefits men and women attain from intergroup encounters. Men likely profit more from engaging in dominance and warfare because it enables them to extract reproductive resources from defeated groups [24], [42]; (see the male warrior hypothesis) [43]. In contrast, women's reproductive interests are perhaps best served by maintaining harmonious intergroup relations (see the tend-and-befriend hypothesis) [35]. Universally, human females tend to lead prosocial nurturing activities such as direct childcare whereas men engage in coalitional aggression activities suggesting that they pursue different intergroup strategies [1], [44].

However, this male-female distinction is dichotomous and does not account for an array of individual variation. Thus, it establishes arbitrary boundaries (i.e., stereotypes) around men and women, and consequently limits what we can determine about gender differences and leadership. A naïve strategy of “if conflict choose a male leader” would be quite limited and not maximize individual differences. On the other hand, phenotypic variations of masculinity and femininity represents a more diagnostic assessment of intergroup leadership potential upon which biological and cultural pressures can select for an optimal context-specific leader (i.e., prototypes). For instance, recent findings indicate a positive relationship between ratings of both male and female facial masculinity and reported dominance [45]. This raises an interesting question of whether followers pay more attention to facial cues of masculinity and femininity over stereotypic difference between male and female when choosing a leader.

### Research Design and Hypotheses

Building on the existing logic of implicit and contingent leadership theories, we first hypothesize that followers will make an initial distinction between the facial cues of a leader and nonleader. Second, we hypothesize that people prefer masculine-looking leaders when intergroup relations are hostile, and, in contrast, feminine-looking leaders are preferred for peaceful intergroup relations. Our final prediction is that the facial cues of masculinity-femininity will be more diagnostic of a leader's perceived qualities than their sex and take precedence. For instance, we expect that *masculine*-females are preferred as leaders over *feminine*-males for war and the converse for peace.

To test this biosocial implicit leadership hypothesis we conducted two experiments on actual and morphed facial images. In the first experiment we used real faces to investigate whether perceptions of leadership in general (i.e., leader/nonleader) and variations of facial masculinity-femininity could successfully predict prototypical preferences for war versus peace leadership. Experiment 1 was with both Western and East Asian samples to provide initial cross-cultural support for these prototypes. In Experiment 2 we manipulated both facial sex cues (male-female) and cues of masculinity-femininity to isolate the signals most influential in predicting the outcome of mock presidential elections. For all experiments written consent was obtained for all participants and the research was approved by the ethics committees from the School of Psychology at the University of Kent and the Department of Social and Organizational Psychology at the VU University Amsterdam.

## Methods

### Experiment 1a

The aim of Experiment 1a was to find evidence for this leader categorization process using Western male faces and a Western sample.

### Participants and Procedure

Thirty-eight participants (21 males, 17 females, *M*_age_ = 22.3, *SD* = 4.6), all students from a university in the United Kingdom, volunteered to complete this pen-and-paper experiment for course credits. They rated black-and-white photographs cropped to remove hair and ears of thirty neutral expression Western male faces on three leadership items ranging from 1 = *not at all* to 7 = *very much*. The items included, “In general, does this person look like a leader?” and “Does this person look like a leader during a time of war [peace]?” Perceived masculinity and femininity of the faces were checked with one item, “Do you rate this person as masculine or feminine?” (1 = *extremely masculine*, 7 = *extremely feminine*). The order of the faces and the scales were counterbalanced. At the end, participants were debriefed, received credits, and thanked for participation.

### Results and Discussion

We used a multilevel analysis for the repeated measurements by treating the within subject ratings as level one and the subjects as level two. The ratings of war and peace leadership were used in two separate analyses as the dependent variable and the ratings of general leadership and masculinity-femininity as predictors of them. To get standardized regression coefficients the ratings were standardized across subjects.

As expected, ratings of general leadership (*β* = 0.45, *p*<.001) and masculinity-femininity (*β* = 0.25, *p*<.001) were significantly associated with perceptions of peace leadership. Likewise, ratings of general leadership (*β* = 0.65, *p*<.001) and masculinity-femininity (*β* = −0.15, *p*<.01) were significantly associated with perceptions of war leadership. It should be noted that the negative coefficient between the ratings of masculinity-femininity and war leadership means that faces rated as more masculine were seen as somewhat more associated with war leadership.

We further checked if the gender of the participant modified these relationships. For war leadership, this was not the case (Gender×General leadership, *p* = .09, Gender×Masculinity-Femininity, *p* = .28). For peace leadership, there was a significant interaction with general leadership (Gender×General leadership, *p*<.01, Gender×Masculinity-Femininity, *p* = .73). The relationship between peace leadership and general leadership was stronger for men than for women. Finally, considering the relationship between masculinity-femininity and general leadership, this was a negative one, meaning that the more feminine a face was seen, the less leader like it was judged to be (*β* = −0.29, *p*<.001). This applied irrespective of the gender of the participant and likely reflects a common association between males and cues of masculinity for leadership when a specific context is not activated.

Overall, however, our results suggest that followers *do* engage in a leadership categorization process of facial perception. Specifically, followership perception of general leadership predicts the likelihood of emergence for both war and peace leadership. Subsequently, it appears context-specific cues of masculinity and femininity then act as contingent factors for respectively assigning war or peace leadership.

### Experiment 1b

Experiment 1b was a replication of Experiment 1a using East Asian faces and an East Asian sample to test the consistency of these leader categorization effects. As a modification to Experiment 1a, we also used female faces to see if the effect generalized across biological sex.

### Participants and Procedure

The sample consisted solely of Indonesian students from an Indonesian university in West Timor and included 46 participants (26 males, 20 females, *M*_age_ = 19.9, *SD* = 2.6). Using pen-and-paper, participants rated 26 photographs of neutral expression Indonesian faces (14 male, 12 female) that were cropped to remove hair and ears. Participants then rated the faces using the same procedure as Experiment 1a only translated into Bahasa Indonesian. Again, participants were debriefed, received credits, and thanked for participation.

### Results and Discussion

The same multilevel analysis procedure utilized in Experiment 1a was applied and we find the same pattern of results to support our hypotheses. Both perceived general leadership (*β* = 0.42, *p*<.001) and perceived masculinity-femininity (*β* = 0.21, *p*<.001) were significant predictors of perceived peace leadership. Similarly, ratings of general leadership (*β* = 0.32, *p*<.001) and ratings of masculinity-femininity (*β* = −0.32, *p*<.001) were significant predictors of perceived leadership during war. Again, the negative coefficient for masculinity-femininity indicates an association between masculine faces and war.

We also checked in this study whether the gender of the participant or gender of the face modified these relationships. For peace leadership, there was a significant interaction for gender of the participant and masculinity-femininity (Gender×Masculinity-Femininity, *p*<.05, all other interactions not significant, .46<*p*-values<.95). The relationship between peace leadership and masculinity-femininity was slightly stronger for men. For war leadership, we found that the gender of the face was a moderator (Gender face×General leadership, *p*<.001, all other interactions not significant, .20<*p*-values<.85). The relationship between war leadership and general leadership was stronger for male faces than for female faces. Looking at the relationship between masculinity-femininity and general leadership, this was dependent on the gender of the face. For male faces it was a negative one, meaning that the more feminine a face was seen, the less leader-like it was judged to be (*β* = −0.11, *p*<.05). For female faces, on the other hand, it was a positive one, meaning that the more feminine a face was seen, the more leader-like it was judged to be (*β* = 0.14, *p*<.05). This applied irrespective of participant gender.

These secondary findings reported in Experiments 1a and 1b are not surprising. They likely reflect a natural artifact in the data regarding average hormonal differences between men and women. Given these differences, it is simply more common for men to be perceived as facially more masculine and women as feminine, and deviating from this average may negatively influence how individual leaders are generally perceived when a specific context is not activating a more discriminatory level of categorization.

In support of our expectations, the primary results further confirm that followers hold an implicit notion of what a leader in general looks like, and this broad perception significantly predicts *both* war and peace leadership emergence. Likewise, at a context-specific level of categorization, this general distinction interacts with specific cues of facial masculinity and femininity to *respectively* assign war and peace leadership. Finally, replication of the results across culture suggests that this implicit leader categorization process is perhaps a commonly shared tool.

### Experiment 2

In Experiment 2 we examined the influence of sex and masculine-feminine facial cues on leader selection as a function of different intergroup relations. To further isolate our variables of interest we morphed both male and female composite images in terms of masculinity and femininity (rather than using unaltered individual faces) and then examined their perceived suitability for intergroup war versus peace leadership in mock presidential elections.

### Pilot

In a pilot we developed an equal number of masculine-male, feminine-male, masculine-female, and feminine-female faces. Twenty participants (10 male, 10 female, *M*_age_ = 26.3, *SD* = 10.6) from a university in the United Kingdom completed the pre-test face ratings. These faces were composed using EFIT-V developed by VisionMetric Ltd. which uses a genetic algorithm to selectively generate facial composites in a desired direction [46]. With this software we “evolved” both masculine and feminine target faces. The facial images were then symmetrized and cropped leaving only a facial mask without hair or ears.

For the pilot, participants were seated in front of a computer monitor and presented 43 faces (11 masculine-male faces, 11 feminine-male faces, 11 masculine-female faces, and 10 feminine-female faces) with one face per slide and asked to rate each face on perceived masculinity-femininity (1 = *extremely feminine*, 7 = *extremely masculine*). Face presentation was counterbalanced. Based on the ratings we selected the 5 most masculine and 5 most feminine scoring faces within each sex to produce a total of 10 pairs (5 male pairs and 5 female pairs) that we used for the experiment. Furthermore, we only selected the faces that were correctly identified as either being a male or female.

### Participants and Procedure

**Figure 2:**
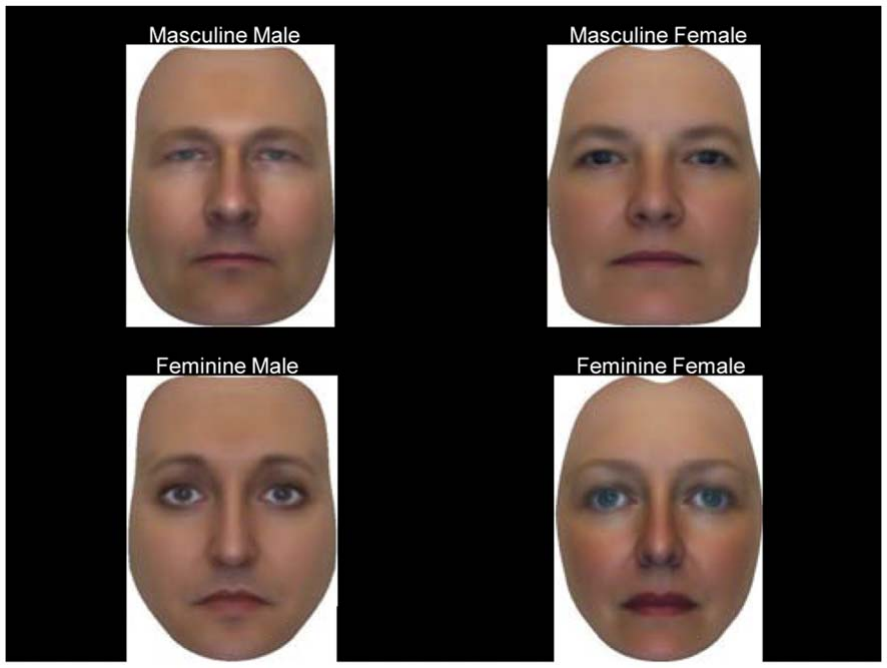
Masculine-feminine face teams: example of stimuli.

One hundred and eighteen participants (57 males, 61 females, *M*_age_ = 24.5, *SD* = 7.6) were recruited and volunteered to complete this online study. The male and female face pairings identified in the pilot were used to create five face teams comprising of one masculine-male, one feminine-male, one masculine-female, and one feminine-female (see Fig. 2).

Two images were presented on the screen at the same time in conjunction with a scenario of a presidential election in a fictitious country (Taminia) during a period when it was having a difficult relationship with a neighboring country. We manipulated the intention to resolve this conflict through dominant (war) or peaceful means. The scenario appeared at the top of the screen with all six pairings of the face teams in a random order in the center of the screen and a “vote” button below each image.

For each scenario the participants were presented with paired combinations of the 4 faces (i.e., six combinations) and then voted for one of the two faces they felt would be most suited as leader in that scenario. Each participant voted twelve times in total (i.e., six pairings for war and six pairings for peace). Presentation of the scenarios, the face teams, and the order of the face pairings were randomized. Upon completion, a debriefing appeared on the screen and participants were thanked for their participation.

### Results and Discussion

To investigate voting preferences for masculine-feminine facial features versus male-female characteristics we conducted analyses within and between scenarios using a Bradley-Terry Model. This particular version of the model accounts for the general preference for the judged objects (masculine-male face, feminine-male face, masculine-female face, and feminine-female face) as well as the interdependency of multiple paired comparisons within the same subject [47]. The general preferences can be reparametrized to yield a 2×2 crossed design of Gender Appearance (masculinity versus femininity of the face), Biological Sex (male versus female faces) and the interaction between these as factors.

As expected, we found that within scenarios only the appearance of masculinity-femininity (not male-female) was a significant factor for both war (Gender Appearance: Wald χ^2^ (df = 1) = 22.11, *p*<0.001; Biological Sex: Wald χ^2^ (df = 1) = 0.24, *p* = .62; Gender Appearance×Biological Sex: Wald χ^2^ (df = 1) = 0.76, *p* = .38) and peace (Gender Appearance: Wald χ^2^ (df = 1) = 26.51, *p*<.001; Biological Sex: Wald χ^2^ (df = 1) = 0.00, *p* = .98; Gender Appearance×Biological Sex: Wald χ^2^ (df = 1) = 3.30, *p* = .07). The estimated effect sizes (odds ratio) were 1.90 (war scenario) and 0.48 (peace scenario), meaning that nearly twice as often a masculine-looking face was chosen for leader in the war scenario, and over twice as often a feminine-looking face was chosen for leader in the peace scenario. Likewise, when investigating patterns between scenarios, only the interaction between masculinity and femininity was significant (Gender Appearance×Scenario: Wald χ^2^ (df = 1) = 48.56, *p*<.001) indicating that differences in voting during war or peace depended solely on masculine-feminine cues (relative to biological sex). The last result can be explained by the fact that the coefficient for Gender Appearance in the two scenarios was nearly the same, only opposite in sign (0.377 and −0.428 for scenarios 1 and 2, respectively). In fact, constraining the coefficient for Gender Appearance to be the same for the two scenarios, only differing in sign, resulted in a nearly as good fitting of a model: Wald Δχ^2^ (df = 1) = 0.34, *p* = .56.

**Figure 3:**
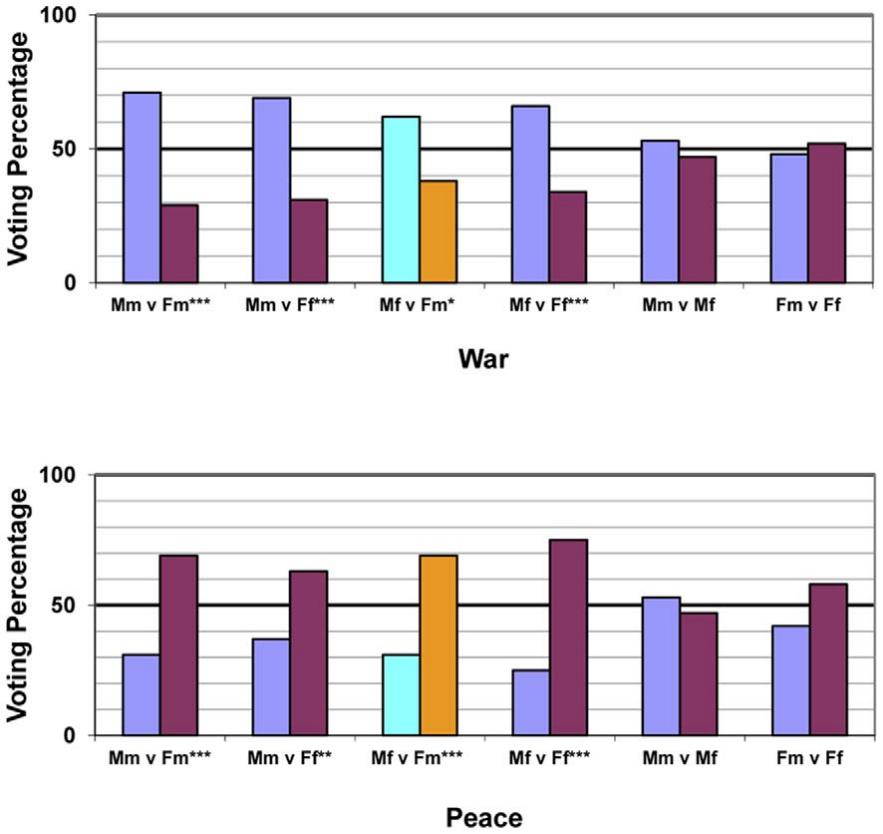
Forced-choice pairs of masculinized and feminized faces during (*A*) intergroup war and (*B*) intergroup peacekeeping. *Note*. M = Masculine, F = Feminine, m = male, and f = female. The highlighted bars represent the Gender Appearance/Biological Sex mismatch (i.e., masculine-female versus feminine-male). *P<0.05, **P<0.01, ***P<0.001.

To present the results in another, more direct way, we averaged the voting responses across all subjects for all possible pairs in both scenarios (6 per scenario), and used chi-squares to evaluate this categorical voting behavior. The individual voting results, displayed in Figures 3*A* and 3*B*, show that for war masculine faces won every pairing unless paired with another masculine face (i.e., masculine-male vs. masculine-female) and conversely the same results for feminine faces during the peace scenario. In addition, as in the model above, the individual voting results also suggest that facial masculinity-femininity is the only influential factor, and sex is not. For instance, the *masculine*-female defeated the *feminine*-male in the war scenario yet the *feminine*-male defeated the *masculine*-female in the peace scenario. In sum, irrespective of their sex, masculine-looking leaders were preferred during war and feminine-looking leaders during peacekeeping.

Again, we considered the gender of the participant as a possible moderator. Neither for the war, nor for the peace scenario was there a significant interaction of the experimental factors with gender (.14<*p*-values<.80), nor a main effect of gender (*p* = .85, *p* = .99, respectively).

## Discussion

The results of two experiments support our biosocial implicit leadership hypotheses. First, an individual's general appearance of leadership predicted whether or not they were likely to be considered for leadership opportunities in both war and peace. Second, facial masculinity and femininity predicted the perceived suitability as either war or peace leader. Third, cues of masculinity-femininity are more influential than actual sex cues at predicting war versus peace leadership. Finally, this categorization process appears to have some consistency across Western and East Asian cultures. Together, our results suggest that subtle facial cues can be used by followers to systematically rank-order leaders versus nonleaders in general and subsequently use context-specific cues to elect leaders depending on how well traits inferred from their facial characteristics match the requirements of the situation. The results have a number of implications.

First, in terms of theory, our research expands both contingency and implicit theories of leadership [9], [15] by showing that environments associated with either intergroup competition or cooperation activate different cognitive leadership prototypes, and individuals who match these prototypes are more likely to emerge as leaders. These previous theories of leadership assumed that such prototypes were learnt but we have argued that they may be evolved decision rules which allow humans to make quick decisions on who they should follow during intergroup relations. Consistent with this, children as young as 5 who are void of political experience can predict the outcomes of elections just by looking at the faces of the candidates [48]. In addition, our findings accord with proximate social identity perspectives on leadership [49]; [50] which suggest that the nature of intergroup relations influences these leadership prototypes.

Second, previous research has found that in unstructured groups men are much more likely to emerge as leaders than women [3], [51]. An implication of our research is that the “think male, think leader” bias [52] may need to be qualified, because our findings suggest that there is a crucial niche for feminine leadership. Both anthropological and primate research highlight these feminine peacekeeping roles [1], [53]. Thus, intergroup cooperation can be an adaptive strategy selecting for feminine leadership styles.

Third, these findings provide compelling evidence for a multi-level process of leadership categorization based on the human face. Finally, it appears that hormonally-based variations in facial masculinity-femininity are more influential in predicting leadership than the male and female distinction. This remarkable observation coincides with evidence that people follow the eye gaze of masculinized faces regardless of the faces' sex [54]. Ultimately, our results promote a classification of potential leaders based on a constellation of masculine and feminine traits, rather than limited stereotypic differences of male and female.

This brings us to note various limitations of our research. We found that preferences for masculine- or feminine-looking leaders shifted as a result of different intergroup contexts, but we did not collect any data about the personality impressions of these leaders based on the facial cues. It is understood that ratings of masculinity are not an exact match with objective sexual dimorphism [55]. Future work should investigate what personality traits correlate with facial masculinity or femininity. Indeed, masculinized male faces are perceived to be more socially and physically dominant [56] which likely interacts with cues of intergroup conflict to influence followership preferences. Another limitation is that for experimental control we used contrived faces (e.g., without hair or ears). Future research may want to use unaltered faces of leader candidates instead. Regarding Experiment 2, due to time and resource constraints it was not possible to exactly replicate using Asian faces on an Asian sample. We acknowledge the potentially unique characteristics of a Western sample [57], though results from Experiment 1 speak to the overall consistency of our findings. Future cross-cultural research may want to consider this paradigm as a method for investigating gender and context-specific leadership. Finally, we examined only political leadership and it would be interesting to find if our results generalize to, for example, business intergroup relations. Our results are in-line with work showing that CEO's with strong faces lead more profitable companies [58].

A practical lesson of our research for aspiring leaders in business and politics is that they should be aware of their image as this affects whether or not they are being judged as a suitable leader. For instance, if a candidate has a more feminine face they are more likely to be selected as leaders when there is a need for peace and internal group cohesion. Another implication for leadership contests is that for feminine-looking individuals it may be advisable to convey messages of intergroup peace, and reconciliation, and masculine-looking individuals should do the opposite and convey tough messages to be persuasive. Whereas previous work suggests that stereotypic perceptions may cause those being observed to act according to the stereotype (i.e., a self-fulfilling prophecy) [59], we provide evidence indicating that it is more advantageous to behave according to one's leadership prototype (i.e., a masculine- or feminine-looking leader) regardless of male-female stereotypes.

To conclude, our research suggests that war and peace elicit different leadership prototypes and that subtle facial cues of aspiring leaders help determine their perceived suitability for the job. As human societies become larger and socially more complex, the physical distance between leaders and followers is likely to increase and as a result indirect visual cues are likely to become more important. Ironically, it seems that our “Stone-Age” leadership experiences still shape our modern “*Face*book-Age” leadership preferences.
